# Policies for primary eye health care in Nigeria: a case study

**Published:** 2022-03-01

**Authors:** Ada Aghaji, Clare Gilbert

**Affiliations:** 1 Professor of Public Health Ophthalmology: College of Medicine, University of Nigeria, Enugu Campus, Nigeria.; 2 Professor of International Eye Health: International Centre for Eye Health, London School of Hygiene & Tropical Medicine, London, UK.

**New primary eye health care policies have the potential to profoundly change how eye health is delivered in Nigeria, provided the gaps are addressed.**

**Figure:**
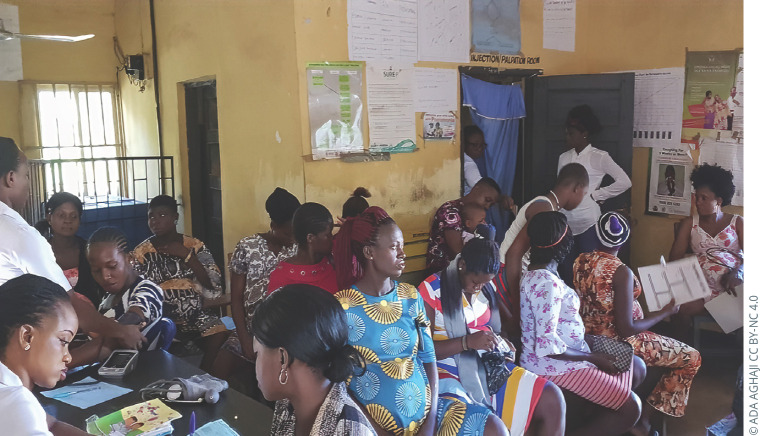
A busy antenatal clinic in a health centre provides an opportunity for eye health promotion amongst pregnant women. **NIGERIA**

Nigeria (population 200 million) has a high prevalence of blindness (0.78%), most of which is avoidable.^1^ The government is increasing political priority for eye health through recent eye health policy statements. The first National Eye Health Policy was launched in February 2022.

We undertook a review of relevant national health policies to identify components which would support the delivery of primary eye health care (PEHC) in Nigeria. Documents were obtained from the Federal Ministry of Health, the Primary Health Care Systems Development Department of the National Primary Health Care Development Agency, and the National Eye Health Strategy team. The policies have several elements of relevance to PEHC (see panel).

## Integrating eye care into primary health care

The National Health Policy (2016) advocates integrating eye care services into existing national health programmes, including PHC, while the National Eye Health Strategic Plan recommends developing a policy to support integrating eye care into PHC.

In addition, a priority area of the National Eye Health Policy is to provide access to equitable eye care, including at the primary level. These supportive policies suggest political priority for integrating eye care into PHC in Nigeria.

## Health work force funding and training at PHC

The Federal Ministry of Health and its parastatals which deliver PHC have policies on human resources for health at PHC level. For example, the National Health Act (2014) states that 10% of national health funding for basic health care should be for training PHC staff.

PHC policies on staffing indicate that the PHC management team should develop a sustainable system for ongoing capacity building of PHC staff. In addition, it is recommended that there should be a minimum number, mix and skill set in each type of PHC facility, and that cadres of workers should deliver services according to their competencies. If the government fully implements these policies, sustainable funding will be available to build capacity of PHC workers who are skilled to deliver a mix of health services, for maternal and child health, mental health, oral health, and eye health, for example.

**National policies in Nigeria that support the delivery of eye care in primary health care (highlights)**

**National Eye Health Strategic Plan (2014–2019)**

- A policy should be developed that includes eye care as an integral part of Primary Health Care (PHC).
- Workers in primary health facilities should undergo training in eye care to identify and manage basic eye conditions.

**NPHCDA Minimum standards for PHC in Nigeria (2015)**

- Chloramphenicol eye drops and ointment, and chlortetracycline ointment, should be available at health centres
- Snellen charts and pen torches should be provided at primary health care centres.

**National Primary Health Care Development Agency: Integrating PHC Governance in Nigeria: PHC under one roof (2016)**

- Primary eye care should be provided to reduce preventable blindness in Nigeria

**National Health Policy (2016)**

- Eye care should be integrated into existing national health programmes

**National Strategic Health Development Plan (2018–2022)**

- Eye care should be included as part of the key non-communicable diseases at primary level
- The package for newborn health should include erythromycin ointment for ocular prophylaxis at all levels

**National Eye Health Policy (2019)**

- Primary health care workers should be trained to provide appropriate eye health services
- Referral forms and registers for eye health should be available across primary and secondary health facilities

## In-service versus pre-service training for eye care

The policies for human resources at PHC level focus on in-service training. Even the Nigeria National Eye Health Strategic Plan recommends in-service training in eye care for PHC workers. It has been suggested that a more effective approach would be to include eye care in the pre-service training of all relevant PHC workers (doctors, nurses, midwives and community health extension workers), possibly as a component of non-communicable diseases training or care of the elderly.^2^ Pre-service training is likely to be less costly than in-service training, and would highlight that eye care is a key component of PHC workers’ role, and not an added responsibility. Refresher in-service training can then be conducted as required. The pre-service training option, which has been successfully implemented for all ages in Rwanda and for children in Bangladesh, would ensure a regular supply of trained staff members, and provide greater coverage and higher quality of care.

## Eye care in health information systems

Currently, the PHC information system does not collect data on eye conditions, as they are not listed as one of the indicators at primary level.^3^ It is expected that the new national eye health policy will address this. Eye health data would be a valuable way of recording and reporting eye health needs in the community, for context-specific planning to appropriately address local eye health needs.

## Policy gaps

Despite the numerous supporting policies for primary eye health care, some policy statements are not consistent across documents. For example, the National Eye Health Policy states that the government will promote quality eye health services at primary, secondary and tertiary levels. However, the National Guidelines for the Development of PHC Systems in Nigeria does not include eye health as a component of PHC, but oral health and mental health are included. Nevertheless, the policy for minimum standards in PHC recommends the provision of basic eye equipment and medication at PHC level. In another example, the national newborn policy recommends the use of topical erythromycin antibiotics to prevent neonatal conjunctivitis, but it is not listed in the essential drugs list for PHC facilities.2 This lack of consistency between policies will need to be addressed by policy makers in the ministry of health and relevant health agencies.

**Figure:**
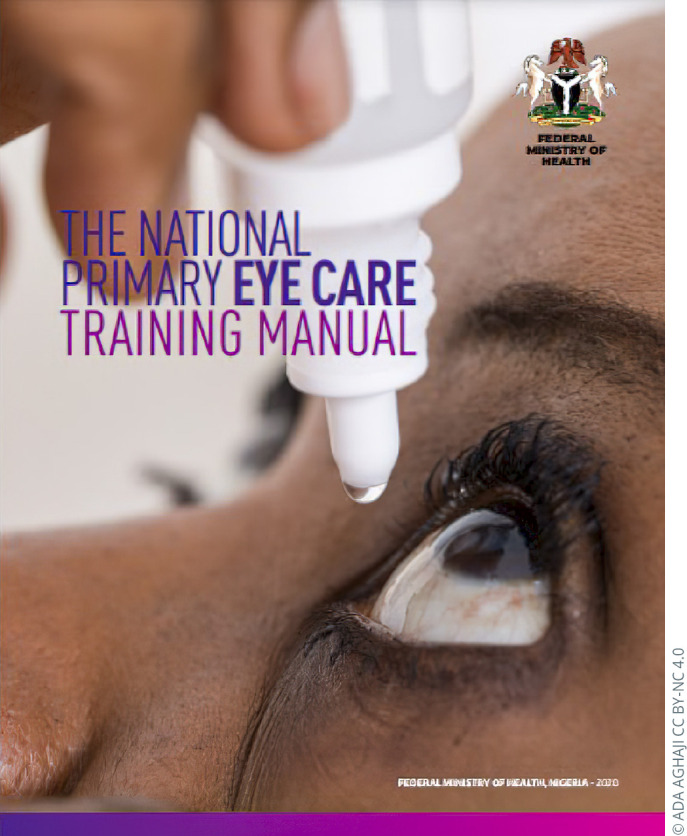
The WHO AFRO primary eye care manual has been adapted for Nigeria by the Ministry of Health. **NIGERIA**

Although primary eye health care in Nigeria has several supportive policies, they are scattered across different policies for PHC, general health and eye health. A clear government policy for primary eye health care as an integral component of PHC which is aligned with other PHC and eye health policies has been recommended.2

Primary eye health care is in its infancy in Nigeria and there is insufficient evidence to indicate what works well and what does not. Nevertheless, if a specific primary eye health care policy were to be developed and implemented, it has the potential to revolutionise how eye care is delivered. Ideally, the policy would cover the following:

- Eye health promotion
- Human resource development, deployment, and retention
- The scope of eye care delivered at primary level
- The provision of appropriate drugs and consumables
- The inclusion of eye conditions in national health information systems and referral mechanisms.
